# Carbonic Anhydrase VI in Skin Wound Healing Study on *Car6* Knockout Mice

**DOI:** 10.3390/ijms21145092

**Published:** 2020-07-18

**Authors:** Toini Pemmari, Jaakko Laakso, Maarit S. Patrikainen, Seppo Parkkila, Tero A. H. Järvinen

**Affiliations:** 1 Faculty of Medicine and Health Technology, Tampere University, 33520 Tampere, Finland; toini.pemmari@tuni.fi (T.P.); jaakko.laakso@tuni.fi (J.L.); maarit.patrikainen@tuni.fi (M.S.P.); seppo.parkkila@tuni.fi (S.P.); 2 Fimlab Ltd., Tampere University Hospital, 33520 Tampere, Finland; 3 Department of Orthopedics and Traumatology, Tampere University Hospital, 33520 Tampere, Finland

**Keywords:** carbonic anhydrase 6, neural growth factor, saliva, breast milk, skin wound, re-epithelization, hypoxia, cell migration, acidification

## Abstract

Carbonic anhydrases (CAs) contribute to tumor cell migration by generating an acidic environment through the conversion of carbon dioxide to bicarbonate and a proton. CA VI is secreted to milk and saliva, and it could contribute to wound closure, as a potential trophic factor, in animals that typically lick their wounds. Our aim was to investigate whether human CA VI improves skin-wound healing in full-thickness skin-wound models. The effect was studied in *Car6* ^−^*^/^*^−^ knockout mice and wild type littermates. Half of both mice strains were given topically administered, milk-derived CA VI after wounding and eight hours later. The amount of topically given CA VI exceeded the predicted amount of natural saliva-delivered CA VI. The healing was followed for seven days and studied from photographs and histological sections. Our results showed no significant differences between the treatment groups in wound closure, re-epithelization, or granulation tissue formation, nor did the *Car6* genotype affect the healing. Our results demonstrate that CA VI does not play a major role in skin-wound healing and also suggest that saliva-derived CA VI is not responsible for the licking-associated improved wound healing in animals.

## 1. Introduction

The healing process of a skin wound consists of four phases: hemostasis, inflammation, proliferation, and remodeling [1]. Among the essential features of the proliferation phase are the migration of keratinocytes and fibroblasts to the wound area and the formation of new blood vessels—i.e., angiogenesis—which also requires cell migration [2]. Growth factors—such as vascular endothelial growth factor (VEGF) and fibroblast growth factor (FGF)—and hypoxic conditions attract keratinocytes and fibroblasts to the wound area and promote angiogenesis [1,2,3]. The lack of oxygen supply shifts cell metabolism toward anaerobic pathways, increasing the production of lactic acid and thus lowering the local tissue pH.

Carbonic anhydrases (CAs) are a large group of enzymes catalyzing the conversion between carbon dioxide and bicarbonate. Through their ability to regulate pH and generate an acidic environment, these enzymes endow tumor cells with survival advantages under hypoxia/acidosis and confer an increased ability to migrate [4]. Many CAs are detected in normal skin [5,6,7], and we showed earlier that the expression of *Car4* and *Car9* mRNAs are elevated in murine skin wounds compared to normal skin during the hypoxic phase of wound healing [8]. This is thought to be associated with the lack of oxygen in the wound milieu [8,9]. Furthermore, topically administered recombinant CA IV accelerates wound closure [8].

Wound licking is a common phenomenon in nature and is intuitive to human beings. Wounds in the oral epithelium, which practically bathe in the saliva constantly, heal quicker than skin wounds. Lack of saliva halts skin-wound healing in rodents [10,11,12,13]. Furthermore, saliva itself promotes wound healing and stimulates re-epithelialization [14]. Hence, saliva must promote wound healing by certain mechanisms that are still inadequately known. Important migration- and angiogenesis-promoting factors in the saliva are epidermal growth factor (EGF) [1] and nerve growth factor (NGF), which accelerate cutaneous wound healing in rodents [15,16], as well as the group of histidine-rich, low-molecular-weight proteins histatins [3]. In addition to these proteins, saliva also contains a CA enzyme, namely CA VI. Thus, saliva could introduce the third CA into the healing skin wound in animals that lick their wounds.

CA VI is the only secreted carbonic anhydrase in mammals. It is present in saliva [17], milk [18], and airways [19,20,21] and may potentially share some physiological properties with NGF [18,22]. As a salivary enzyme, CA VI affects taste perception [23,24], protects the mucosa of esophagus [25], and normalizes taste bud architecture [23]. However, CA VI is not expressed in the normal skin-stratified epithelium or skin wound [8], but being a salivary protein, CA VI could be easily placed on healing wounds via licking. It has the ability to regulate tissue acid–base homeostasis, and it has also been proposed as a growth-promoting factor [26]. We recently discovered that another CA enzyme—CA IV—plays a role in skin-wound healing, while CA VI was not studied as it was not among two CA enzymes endogenously expressed in the skin wound [8]. Since the exact molecular mechanism by which saliva influences wound healing is not known, we wanted to investigate if CA VI isolated from human milk shows any potential therapeutic role in skin-wound healing using a murine full-thickness excisional skin-wound model and CA VI knockout mice (*Car6*^−^*^/^*^−^).

## 2. Results

### 2.1. Sequence Identity between CA VI and NGF

We analyzed the sequence identity between CA VI and NGF using Basic Local Alignment Search Tool (BLAST) Global Align search. Since the plan was to treat mice (*Mus musculus*) with human (*Homo sapiens*) CA VI, both species were analyzed. The sequence identity analysis revealed that human CA VI and mouse CA VI share identity of 51%. Comparisons between NGF and CA VI among or between species showed less than 20% identity (Table 1).

### 2.2. CA VI Treatment in Skin Wounds

**Figure 1:**
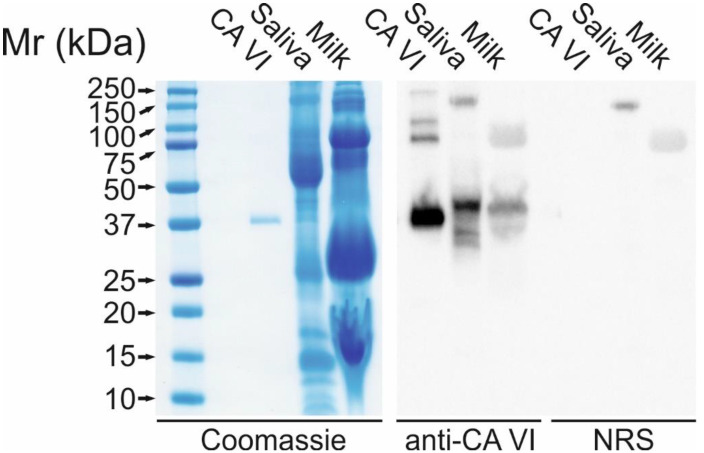
SDS-PAGE and Western blot of purified human CA VI (0.5 ug), saliva (10 uL), and milk (10 uL) using Colloidal Coomassie Blue staining or immunodetection by 1:1000 diluted rabbit anti-human CA VI serum. Normal rabbit serum (NRS, 1:1000 dilution) was used for controls. In SDS-PAGE, the purified CA VI appears as a 40 kDa polypeptide. Western blot shows slightly higher molecular weight bands (42–44 kDa) in both saliva and milk. The slight variation in electrophoretic mobility is probably due to different glycosylation states of the proteins. In addition, the purified CA VI protein shows several higher molecular weight polypeptides in Western blot, which may be attributed to the tendency of CA VI to form oligomeric species.

**Figure 2:**
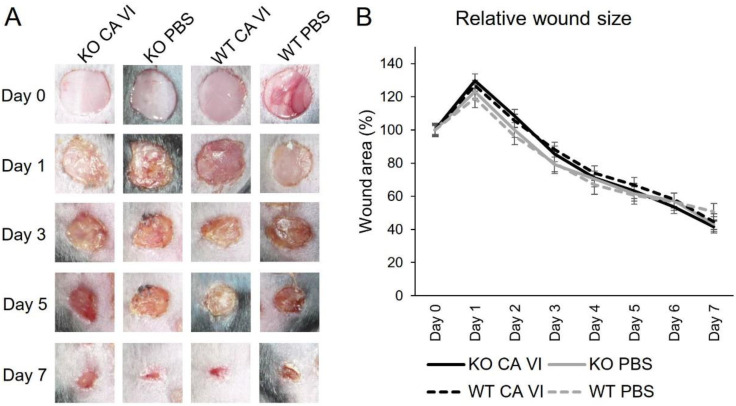
The purified CA VI and macroscopic analysis of the wounds. (**A**) Examples of skin wounds at different timepoints. (**B**) The wound sizes relative to the size on the wounding day (day 0) were calculated from the measurements of digital photographs. Error bars represent SEM, *n* = 8. No statistically significant difference was observed by analysis of variance.

We examined the effects of the lack of endogenous CA VI and exogenously administered human CA VI on murine skin-wound healing. First, we generated CA VI knockout (*Car6*^−^*^/^*^−^) mice. These mice are fertile and develop normally [27]. Then highly active CA VI enzyme, in turn, was isolated and purified from human milk essentially as described previously [28,29] (Figure 1). *Car6*^−^*^/^*^−^ (KO) and wild-type (WT) mice were divided in two groups receiving topically either CA VI or saline (control) in poloxamer 407 gel. The first dose was given right after wounding and the second dose eight hours later. The daily examination of macroscopic wound size revealed no differences between the treatment groups (Figure 2).

**Figure 3:**
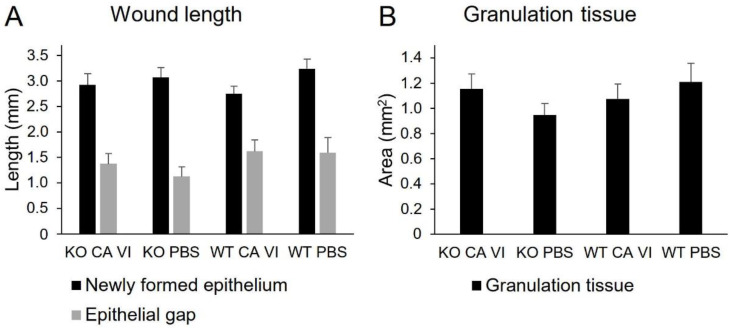
The microscopic analysis of the wounds. (**A**) The length of newly formed epithelium (black) and epithelial gap (gray). (**B**) The area of granulation tissue. Error bars represent SEM, *n* = 6 CA VI KO, *n* = 7 wild-type (WT) phosphate-buffered saline (PBS), *n* = 8 others. No statistically significant differences were found by analysis of variance.

After seven days follow-up, the mice were sacrificed for histological analysis. There was no significant difference in the length of newly formed epithelium (epidermal tongues), in the length of the gap between the new epithelial tongues, or in the size of the granulation tissue (Figure 3). In addition, the number of open and closed wounds was not significantly different between the groups (Table 2).

## 3. Discussion

Despite the hypoxia prevailing in skin-wound tissue until the vascular supply of oxygen is re-established by angiogenesis around day 5, the effects of pH-regulating enzymes, carbonic anhydrases, on skin-wound healing have not been addressed until recently [7,8]. In our previous study, we studied the expression pattern of 11 family members of CAs [8]. We demonstrated that the expression of two CA family members—*Car4* and *Car9* genes—was upregulated in skin wounds, peaking on days two and three [8]. Furthermore, topically administered recombinant CA IV enzyme accelerated wound closure [8]. In the current study, we wanted to see if the secretory enzyme CA VI—which could be brought into skin wounds by animals that lick their wounds—influences skin-wound healing. Using the full-thickness skin wound model, we could not demonstrate any retarded wound healing phenotype in *Car6^-/-^* mice, nor did we see any enhancement in wound healing when treated with exogenous human CA VI enzyme.

Wound-licking is a common behavior among animals. It is also generally known that wounds in the oral epithelium, which are constantly covered with saliva, heal quicker than skin wounds. Furthermore, lack of saliva halts skin-wound healing in rodents, while saliva itself stimulates wound closure and re-epithelialization [10,11,12,13,14]. Hence, saliva must promote wound healing by certain mechanisms that are still inadequately known. To understand the effects of saliva on wound healing, we focused on CA VI enzyme, which is abundantly present in both saliva and milk [18]. CA VI has the ability to regulate local pH [25,30,31]. In addition to that, some of its physiological properties may potentially be similar to NGF [18,22], and thus it has been postulated that CA VI could have growth-factor-like properties [26,32]. Therefore, we hypothesized that CA VI may alter wound healing via pH modulation of the extracellular milieu or via growth factor activity like NGF. NGF increases the migration of keratinocytes and the tube formation of endothelial cells in vitro [33] and accelerates skin-wound healing in rodents [15,16]. Based on our sequence analysis (Table 1), the homogeneity between NGF and CA VI is very low, and it seems unlikely that CA VI could act as a ligand for the NGF receptors.

The amount of exogenous CA VI used in this study was higher than its concentration in saliva [34,35,36], but we only provided a fraction (approx. 25%) of the amount of recombinant CA IV used in our previous study [8]. Human CA VI enzyme was selected for this study as large quantities of high-quality human CA VI can be easily isolated from human milk for translational treatment trial in mouse. However, the human CA VI has a disadvantage when tested in mouse; the identity between human and mouse CA VI enzymes is quite low (Table 1). This may have influenced the outcome in our trial, especially if the effects of CA VI depend on protein–protein interactions and thus require certain structural conformation.

Both endogenously expressed CAs in the wound—CA IV and CA IX—show remarkable restricted expression in the wound [8]. They form a band-like expression pattern just underneath the eschar [8]. Taking together the temporal expression of CA IV and IX during the hypoxic phase of wound healing and the restricted location within the wound [8], these findings implicated that CAs generate a very specific acidic microenvironment within the hypoxic wound and provide selective migration benefit for migrating keratinocytes as they close the gap in the skin wound [8]. Interestingly, although the mRNA expression of *Car4* and *Car9* genes peaks between days 2 and 3 in skin wounds, the expression of both CA IV and IX enzymes peaks substantially later, between days 5 and 10 [8]. This is somehow striking as days 5–7 is the time point when angiogenesis has already peaked in skin wounds [37,38]. As the sprouting angiogenesis starts from the bottom of the wound, this indicates that hypoxia still persists on the top of the wound—i.e., underneath the scab tissue, where keratinocytes migrate and where CA IV and IX are expressed [8]—and long duration of site-specific CA enzyme activity is in demand during skin-wound healing [8]. For therapeutic purposes, this suggests that sustainable delivery and retainment of CAs are needed to enhance tissue regeneration. As the scab formed at the wound essentially seals the healing wound from its environment, this may indicate that the CA VI concentration in situ was not high enough for its physiological function in either pH modulation or tissue growth.

## 4. Materials and Methods

### 4.1. Blast Analysis

The Global Alignment tool (Needleman-Wunsch) [39] of Basic Local Alignment Search Tool (BLAST) [40] was used to compare the sequence similarities. Gap Cost parameter was set to existence 11 and extension 2. The FASTA sequences for mouse CA VI (sp|P18761|CAH6_MOUSE), human CA VI (sp|P23280|CAH6_HUMAN), mouse NGF (sp|P01139|NGF_MOUSE), and human NGF (sp|P01138|NGF_HUMAN) were acquired via UniProt database [41].

### 4.2. Reagents

CA VI protein was extracted from human breast milk donated by breastfeeding mothers at Tampere University Hospital. The extraction method of CA VI has been published earlier [28], and purification was conducted twice to increase the amount of purified protein. Poloxamer 407 was purchased under tradename Pluronic F-127 from Sigma (St.Louis, MO, USA). The rest of the chemicals were purchased from Sigma unless otherwise stated.

### 4.3. Western Blot and SDS Polyacrylamide Gels

Purified protein was verified by SDS polyacrylamide gel (SDS-PAGE) and Western blotting. Purified human CA VI (0.5 μg) in 20 μL running buffer, 10 μL human saliva, and 10 μL human breast milk were run on Any kD Mini-PROTEAN TGX Stain-Free Protein (Bio-Rad, Hecules, CA, USA) gels, 200 V for 30 min. One gel was stained with PageBlue Protein Staining Solution (Thermo Fisher, Waltham, MA). The other gel was transferred to Trans-Blot Turbo Mini 0.2 μm PVDF Transfer Packs (Bio-Rad) membrane with BioRad’s Trans-Blot Turbo Transfer System (3 min). The membrane was blocked with TBS Blotto A (Santa Cruz Biotechnology, Dallas, TX, USA) for 30 min at room temperature. Rabbit anti-human CA VI serum [42] or normal rabbit serum (control), both diluted 1:1000, was incubated overnight at 4 °C. The secondary anti-rabbit IgG (#R1364HRP, Acris antibodies, Rockville, MD, USA) diluted 1:20,000 was incubated for 45 min at room temperature. After both incubations, the membrane was washed four times. The Western blot was visualized with WesternBright (Advansta, San Jose, CA, USA) according to the manufacturer’s instructions.

### 4.4. Mice

The *Car6^-/-^* generation and phenotype are described previously [27]. Sixteen female knockout *Car6^-/^*^-^ and 16 female wild-type control littermates were included in the study. The mice were 11 weeks old and weighed 18–26 g at the time of wounding. The mice were housed individually after wounding and fed by standard laboratory pellets and water ad libitum.

The wound model was accepted by the National Animal Ethics Committee of Finland on 12 September 2017 (ESAVI/6422/04.10.07/2017).

### 4.5. Wound Model

After analgesia with buprenorphine (0.15 mg/kg), each mouse was anesthetized with 4% isoflurane (carrier gas 0.2 mL/min oxygen and 0.4 mL/min room air), and the anesthesia was maintained with 2% isoflurane. The lower back skin of the mouse was shaved and disinfected with 70% ethanol. Four circular (diameter 6 mm) wounds were marked with a biopsy punch and cut with scissors [38]. After that, 60 μg human CA VI diluted in 140 μL phosphate-buffered saline (PBS) or 140 μL plain PBS, mixed with 60 μL ice-cold poloxamer 407, was pipetted evenly on the four wounds and left uncovered [8]. When the poloxamer 407 was hardened, the animal was transferred to a 37 °C warm environment and, when fully recovered, back to its own cage.

Eight hours after wounding, the mice were anesthetized as described above. A second dose of either 60 μg human CA VI diluted in 140 μL PBS or 140 μL plain PBS, mixed with 60 μL ice-cold poloxamer 407, was pipetted evenly on the wounds. After the solution had hardened, the animal was allowed to recover as described above.

The wounds were photographed with a digital camera daily. For the photographing, the mice were anesthetized as described above. On day 7 after wounding, the animals were sacrificed with carbon dioxide and cervical dislocation after photographing. The back skin, including subcutaneous tissue and topmost layers of the superficial back muscles containing the wounds, was removed with a scalpel.

### 4.6. Wound Closure

The wound photographs were analyzed blinded with ImageJ (NHS, Bethesda, MD, USA) [43]. The edges of each wound were drawn, and the enclosed area was recorded. Each photograph included a control square to determine the actual sizes of the wounds.

### 4.7. Histology

The back skin removed after anesthesia was placed on a piece of PVDF Transfer membrane (Hybond-P, Amersham Biosciences, Amersham, UK) to keep it straight. The tissue was fixed with 4% paraformaldehyde in PBS for 24 h. After that, the sample was rinsed twice with 70% ethanol, cut in four pieces, and embedded in paraffin. The paraffin-embedded sections were cut and stained with hematoxylin–eosin (VWR, Radnor, PA, USA).

The hematoxylin–eosin-stained sections were analyzed blinded with Aperio ImageScope (Leica biosystems, Chicago, IL, USA) [44]. The lengths of the newly formed epithelium and the epithelial gap as well as the area of granulation tissue were measured from the sections. Whenever possible, two measures were taken from one wound and averaged. The number of closed wounds (a continuous epithelial over the wound site) and open wounds were calculated. A wound was considered open if there was an epithelial gap in either of the two measures.

### 4.8. Statistical Analysis

IBM SPSS Statistics version 24 (IBM, Armonk, NY, USA) was used for the statistical analysis. The normality of each variable was tested with Shapiro–Wilk and, based on the result, either one-way analysis of variance or Kruskall–Wallis test was used. The amount of open and closed wounds was analyzed with chi-squared test. *p*-values under 0.05 were considered significant.

## 5. Conclusions

Many animals lick their wounds if allowed. Licking is considered a mechanism to clean the wound and get rid of potentially harmful bacteria, but recurrent licking makes the scab thinner, and saliva also contains proteins that could enhance the actual regenerative response after wounding. We have assessed in this manuscript the regenerative potential of CA VI enzyme, which is abundant in saliva, by utilizing both *Car6*^−^*^/^*^−^ mice and isolated human CA VI enzyme. Our results demonstrate that CA VI does not play a major role in skin-wound healing and suggest that saliva-derived CA VI is not responsible for the licking-associated improved wound healing in animals.

## Figures and Tables

**Table 1:** The number of identities, positives, and gaps between the carbonic anhydrase VI (CA VI) and nerve growth factor (NGF) in mouse (*Mus musculus*) and human (*Homo sapiens*). Each pair is aligned with BLAST Global Align tool. Identities refer to same amino acids, positives refer to amino acids having similar physico-chemical properties, and gaps refer to spaces introduced to compensate insertions and deletions.

Comparison	Identities		Positives		Gaps	
human CA VI mouse CA VI	162/320	(51%)	220/329	(68%)	15/320	(4%)
human CA VI mouse NGF	57/317	(18%)	93/317	(29%)	85/317	(26%)
human NGF human CA VI	55/315	(17%)	89/315	(28%)	79/315	(25%)
mouse NGF mouse CA VI	50/320	(16%)	98/320	(30%)	82/320	(25%)

**Table 2:** The number of closed (complete re-epithelialization) and open wounds on day 7 based on histological analysis. *n* = 7 CA VI KO, *n* = 8 others. No statistically significant differences in chi-squared analysis.

Wound Status	KO CA VI		KO PBS		WT CA VI		WT PBS	
Closed	5	(21%)	10	(31%)	6	(19%)	9	(33%)
Open	19	(79%)	22	(69%)	25	(81%)	18	(67%)

## Abbreviations

BLAST	Basic Local Alignment Search Tool
CA	Carbonic anhydrase
Car	Mouse carbonic anhydrase gene
EGF	Epidermal growth factor
FGF	Fibroblast growth factor
KO	Knockout
NGF	Nerve growth factor
NRS	Normal rabbit serum
PBS	Phosphate-buffered saline
SEM	Standard error of the mean
VEGF	Vascular endothelial growth factor
WT	Wild type
